# Age-Related Neurodevelopmental Patterns of Visuospatial Attention Deficits in Children and Adolescents with ADHD: ERP Evidence for Late-Stage Cognitive Control Impairment

**DOI:** 10.3390/biology15141213

**Published:** 2026-07-22

**Authors:** Enguo Wang, Xin Zhang, Yimin Ma

**Affiliations:** 1 Center for Mental Health Education and Counseling, Zhengzhou Health College, Zhengzhou 475004, China; 2 Faculty of Education, Henan University, Kaifeng 475100, China

**Keywords:** ADHD, visuospatial attention, cognitive load, cross-sectional age differences, executive control, event-related potential (ERP), P3

## Abstract

Attention-Deficit/Hyperactivity Disorder (ADHD) is a neurodevelopmental disorder characterized by inattention, hyperactivity, and impulsivity. This study used high-temporal-resolution event-related potentials (ERPs) to investigate visuospatial attention allocation across early perceptual and late cognitive control stages in 62 children and adolescents with ADHD and 62 typically developing (TD) controls (total N = 124), using a dual-task paradigm under a varying cognitive load. Behaviorally, the ADHD group showed significantly lower accuracy and marginally prolonged reaction times relative to TD controls. Electrophysiologically, the P2 component exhibited significant age effects, with shorter latency in the ADHD group than in the TD group under high load. N2 showed no significant group differences. The P3 component displayed significantly enhanced amplitude and prolonged latency in the ADHD group, especially under high load, indicating impaired late-stage cognitive control and working memory maintenance. These findings reveal that high-symptom (ADHD-screened) visuospatial attention deficits are more pronounced during late cognitive control stages; however, early perceptual processing also shows load-dependent alterations, with P3 abnormalities may represent candidate neural correlates of late-stage cognitive control deficits, although their clinical utility requires further evaluation. These results clarify the age-related patterns of attentional dysfunction in children and adolescents with ADHD and provide electrophysiological evidence for its neural mechanisms.

## Simple Summary

Children and teens with Attention-Deficit/Hyperactivity Disorder often struggle to focus on visual information, yet scientists still do not know whether their attention problems start when they first see images or when they make active mental decisions. This study recruited 124 young people aged 7 to 14, including 62 children with ADHD and 62 typical developing peers. We used a dual-task test with two levels of mental difficulty and brain wave recording to track two stages of visual attention. When mental tasks were hard, young people with ADHD made more mistakes and reacted slower. Their early visual brain signals only showed small differences, while later brain signals linked to active thinking were obviously abnormal, especially for younger kids. These brain differences weakened as teenagers grew older. This work indicates that core attentional deficits associated with ADHD are most prominently expressed during late-stage cognitive control, rather than basic visual perception. The brain wave marker found here can help doctors better judge ADHD and design targeted training to improve children’s concentration.

## 1. Introduction

Attention-Deficit/Hyperactivity Disorder (ADHD) is a neurodevelopmental disorder defined by developmentally inappropriate inattention, hyperactivity, and impulsivity that impairs daily functioning across academic, social, and familial domains [1]. Core difficulties include sustained attention deficits, distractibility, poor response inhibition, and dysregulated motor activity, with onset typically before 6 years of age [2]. The DSM-5 categorizes ADHD into three presentations: predominantly inattentive, predominantly hyperactive-impulsive, and combined [1]. While early diagnosis is challenging due to overlapping typical developmental behaviors, most cases are identified between 7 and 12 years when academic demands intensify, and approximately 70% of affected individuals continue to exhibit symptoms into adolescence, often comorbid with learning difficulties, emotional problems, and interpersonal dysfunction [3].

Visuospatial attention is a foundational cognitive system supporting stimulus selection, resource allocation, and goal-directed processing. Deficits in this domain are central to ADHD psychopathology, manifesting as inefficient filtering of irrelevant inputs, reduced attentional control, and impaired working memory maintenance [4] Contemporary research has shifted from behavioral and questionnaire-based assessments to neurophysiological techniques—especially event-related potentials (ERPs)—which afford millisecond-scale temporal resolution to dissect the temporal stages of attentional processing. ERP components, including P2, N2, and P3, index distinct processing stages: P2 reflects early perceptual encoding and sensory gating; N2 indexes response conflict monitoring and inhibitory control; and P3 signals late-stage cognitive control, working memory updating, and central–parietal attentional resource allocation [5,6].

Accumulating ERP evidence indicates atypical neural activity in ADHD during attentional tasks. Children and adolescents with ADHD show altered N2 and P3 amplitudes, prolonged P3 latency, and atypical frontoparietal network activity, consistent with impaired executive control and resource distribution [7]. However, critical gaps remain: most studies do not dissociate early perceptual versus late cognitive control contributions to visuospatial attentional dysfunction, nor do they examine cross-sectional age-related differences across childhood and early adolescence under varying cognitive load. Although cross-sectional findings suggest age-related improvements in attention, it remains unclear whether screening-identified ADHD group deficits arise from delayed maturation or stable, trait-like neural dysregulation [8,9]. Furthermore, few studies use graded-load dual-task paradigms to simulate real-world cognitive demands, limiting ecological validity [10].

To address these limitations, this study employed a high-temporal-resolution ERP approach combined with a dual-task paradigm manipulating cognitive load, to characterize visuospatial attentional processing in children and adolescents aged 7–14 years. We compared ADHD and typically developing (TD) groups across three age bands to examine age-related differences in early perceptual and late cognitive control stages. Based on prior theory and evidence, we propose two hypotheses: First, both ADHD and TD groups will show higher accuracy and faster reaction times under low cognitive load than under high cognitive load. Second, ADHD-related visuospatial attention deficits will localize primarily to late-stage cognitive control processes, as indexed by abnormal P3 amplitude and prolonged latencies, particularly under high-load conditions, with minimal between-group differences in early perceptual (P2) and intermediate inhibitory (N2) stages.

## 2. Method

### 2.1. Participants

This study was approved by Institutional Review Board of Henan Provincial Key Laboratory of Psychology and Behavior (protocol code 20240705002 and date of approval 5 July 2024). From an initial screening of 4347 students aged 7–14 years at an urban elementary school, participants were assessed using the Conners Parent Symptom Questionnaire (PSQ) and the Swanson, Nolan, and Pelham-IV Rating Scale (SNAP-IV, Parent Version). Inclusion criteria were as follows: right-handedness, normal or corrected-to-normal vision, no history of neurological disorders, epilepsy, brain injury, or other psychiatric disorders, and no current psychotropic medication use. Children with a PSQ Z-score ≥ 1.5 and SNAP-IV total score > 1.1 were assigned to the ADHD group, while those with a PSQ Z-score < 1.5 and SNAP-IV total score < 1.0 formed the typically developing (TD) control group. The final sample comprised 62 children with ADHD (45 males, 17 females; mean age = 10.58 ± 2.11 years) and 62 TD controls (47 males, 15 females; mean age = 10.72 ± 1.95 years), totaling 124 participants. To examine cross-sectional age-related differences, participants were divided into three non-overlapping age bands: 7–9, 10–12, and 13–14 years. Written informed consent was obtained from parents or legal guardians, and assent was obtained from all children. The demographic characteristics of the participants are shown in Table 1.

### 2.2. Research Instruments

#### 2.2.1. Conners Parent Symptom Questionnaire (PSQ)

The PSQ is a 48-item parent-rated questionnaire (0–3 scale) used to assess hyperactivity, inattention, and conduct problems. The Chinese version has good reliability and validity (Cronbach’s α = 0.92) [11]. A Z-score ≥ 1.5 was used as the cutoff for ADHD screening.

#### 2.2.2. SNAP-IV Parent Version

The 26-item SNAP-IV is used to assess inattention, hyperactivity–impulsivity, and oppositional symptoms (0–3 Likert scale). The Chinese version shows excellent internal consistency (α = 0.95) [12].

#### 2.2.3. Visuospatial Dual-Task Paradigm

A visuospatial dual-task paradigm was used to manipulate cognitive load (low vs. high). Each trial began with a central fixation cross (800 ms), followed by a stimulus array consisting of an arrow and digits presented for 3000 ms.

**Figure 1:**
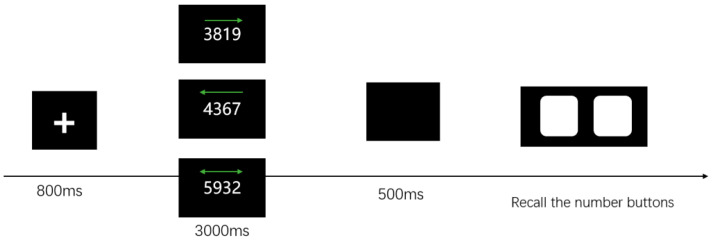
Flowchart for dual-task stimulus presentation.

Under low load, participants remembered one digit at each end of the arrow (total of two digits); under high load, they remembered two digits in the direction indicated by the arrow. After a 500 ms blank screen, participants recalled the digits orally or in writing. To ensure attention to the load condition, participants additionally pressed ‘J’ for low-load trials and ‘F’ for high-load trials as a secondary task. The practice block included 10 trials, followed by a formal test of 180 trials (90 low load, 90 high load, randomized), with 45 trials per load condition for each visual field (left/right). A flowchart of the stimulus presentation is provided in Figure 1.

### 2.3. EEG Acquisition and Preprocessing

EEG was recorded using a 64-channel EGI system at a 1000 Hz sampling rate, with all electrode impedances kept below 5 kΩ. The online reference was set to Cz, and the data were offline re-referenced to the average of linked mastoids. Preprocessing was performed in MATLAB R2020b and included band-pass filtering (0.1–30 Hz), epoching from −200 to 800 ms relative to stimulus onset, baseline correction (−200 to 0 ms), artifact rejection (threshold ±100 μV), independent component analysis (ICA) for eye-blink correction, and interpolation of bad channels. ERP components were quantified at specific electrode clusters: P2 (190–270 ms) at frontal–central sites (AFz, Fz, FCz, F1, F2, FC1, FC2); N2 (180–280 ms) and P3 (350–600 ms) at central–parietal sites (Cz, CPz, Pz, C1, C2, CP1, CP2). This central–parietal selection follows standard practice for attention- and executive-related ERP components, as occipital sites are primarily used for visual evoked potentials.

### 2.4. Data Acquisition and Analysis

Statistical analyses were performed using SPSS 27.0. A 2 (Group: ADHD, TD) × 2 (Load: low, high) × 3 (Age: 7–9, 10–12, 13–14 years) repeated-measures analysis of variance (ANOVA) was conducted on behavioral accuracy, reaction times, and the mean amplitudes and latencies of the P2, N2, and P3 components. Follow-up independent-sample *t*-tests and post hoc pairwise comparisons were performed as appropriate. Significance was set at *p* < 0.05. Effect sizes are reported as partial eta-squared (*η_p_*^2^). Degrees of freedom were corrected using the Greenhouse–Geisser method where necessary, and the total sample size (N = 124) was used for all analyses.

For all post hoc pairwise comparisons, Bonferroni correction was applied. Specifically, for comparisons involving the three age groups (7–9, 10–12, 13–14 years), the adjusted significance threshold was set at *p* < 0.0167 (0.05/3); for comparisons between the two load conditions (low vs. high), the adjusted threshold was set at *p* < 0.025 (0.05/2). In the Section 3, we report the Bonferroni-adjusted *p*-values for all post hoc comparisons, and statistical significance is determined based on these adjusted thresholds.

## 3. Results

### 3.1. Behavioral Results

#### 3.1.1. Accuracy

Group main effect: *F* (1,122) = 6.140, *p* = 0.014, *η_p_*^2^ = 0.041, ADHD accuracy (0.867) < TD (0.914). Load main effect: *F* (1,122) = 71.197, *p* < 0.001, *η_p_*^2^ = 0.331, high load (0.861) < low load (0.919). Age main effect: *F* (2,122) = 18.236, *p* < 0.001, *η_p_*^2^ = 0.202. Load × Age interaction: *F* (2,122) = 9.700, *p* < 0.001, *η_p_*^2^ = 0.119. Three-way interaction: not significant.

#### 3.1.2. Reaction Time

**Figure 2:**
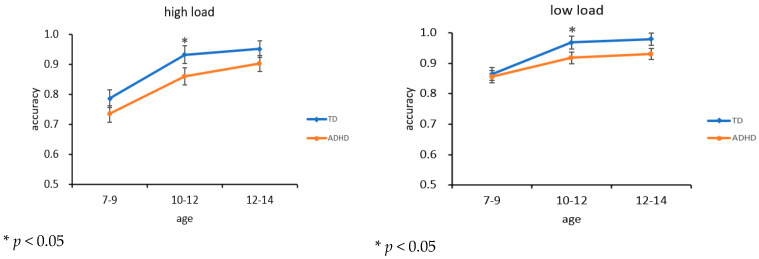
Line plots of correctness for two groups of subjects of different ages. Error lines represent standard errors.

**Figure 3:**
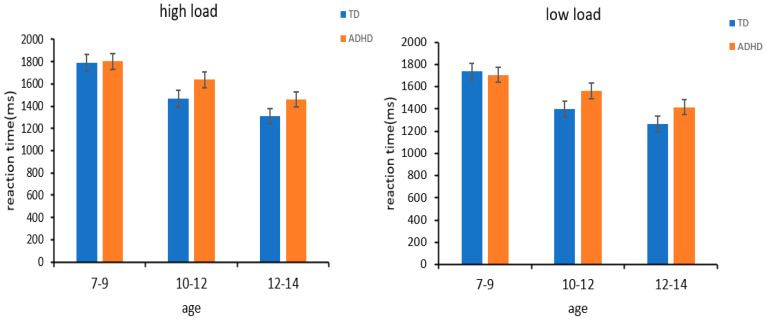
Reaction timeline plots for the two groups across age subgroups. Error bars represent standard errors.

Load main effect: *F* (1,122) = 44.509, *p* < 0.001, *η_p_*^2^ = 0.236, low load faster (1513.21 ms) than high load (1578.72 ms). Age main effect: *F* (2,122) = 16.631, *p* < 0.001, *η_p_*^2^ = 0.188. Group effect: marginally significant *F* (1,122) = 3.342, *p* = 0.070, *η_p_*^2^ = 0.023, ADHD RT (1597.71 ms) longer than TD (1494.22 ms). The behavioral results for accuracy and reaction time are illustrated in Figure 2 and Figure 3.

### 3.2. Electrophysiological Results

A 2 (Group: ADHD, TD) × 2 (Load: low, high) × 3 (Age: 7–9, 10–12, 13–14 years) repeated-measures ANOVA was conducted on the amplitude and latency of P2, N2, and P3, followed by post hoc tests and independent-sample *t*-tests (Figure 4, Figure 5, Figure 6, Figure 7, Figure 8, Figure 9, Figure 10 and Figure 11).

#### 3.2.1. P2 Component

For P2 amplitude, no significant main effects of the group or load, or interactions involving the group, were found (all *p* > 0.05). For P2 latency, a significant main effect of the load was observed, with a shorter latency under high load, *F* (1,122) = 10.24, *p* = 0.002, *η_p_*^2^ = 0.078; a significant main effect of age also emerged, with latency decreasing with age, *F* (2,122) = 21.56, *p* < 0.001, *η_p_*^2^ = 0.264. Critically, a significant Group × Load interaction was detected, *F* (1,122) = 6.87, *p* = 0.010, *η_p_*^2^ = 0.053. Follow-up *t*-tests revealed that under high load, P2 latency was significantly shorter in the ADHD group than in the TD group (*t* (122) = 2.41, *p* = 0.017), whereas no group difference was found under low load (all *p* > 0.05).

#### 3.2.2. N2 Component

No significant main effect of the group or load was found for N2 amplitude or latency (all *p* > 0.05). A significant main effect of age was observed for latency, which shortened with age, *F* (2,122) = 8.32, *p* = 0.001, *η_p_*^2^ = 0.121. No significant Group × Load, Group × Age, or three-way interaction was found for either amplitude or latency (all *p* > 0.05). Independent-sample *t*-tests confirmed no significant group differences in N2 metrics under either load condition or in any age subgroup (all *p* > 0.05).

#### 3.2.3. P3 Component

P3 amplitude showed a significant main effect of the group, with a larger amplitude in the ADHD group, *F* (1,122) = 7.92, *p* = 0.006, *η_p_*^2^ = 0.062; a significant main effect of the load was also present, with a larger amplitude under high load, *F* (1,122) = 52.68, *p* < 0.001, *η_p_*^2^ = 0.304. For P3 latency, significant main effects of group and load were found: the ADHD group exhibited longer latency, *F* (1,122) = 9.15, *p* = 0.003, *η_p_*^2^ = 0.070, and latency was prolonged under high load, *F* (1,122) = 47.31, *p* < 0.001, *η_p_*^2^ = 0.282. Latency decreased significantly with age, *F* (2,122) = 19.47, *p* < 0.001, *η_p_*^2^ = 0.245.

Most importantly, significant Group × Load interactions were observed for both P3 amplitude and P3 latency. For P3 amplitude, the Group × Load interaction was significant, *F* (1,122) = 5.318, *p* = 0.023, *η_p_*^2^ = 0.040; for P3 latency, the interaction was also significant, *F* (1,122) = 5.978, *p* = 0.016 *η_p_*^2^ = 0.044, indicating that group differences in both measures were more prominent under high load. A significant Group × Age interaction was also found, *F* (2,122) = 4.26, *p* = 0.016, *η_p_*^2^ = 0.066. Under high load, the ADHD group showed significantly larger P3 amplitude and longer latency than the TD group (*t* = 2.78, *p* = 0.007; *t* = 3.02, *p* = 0.003). Age-stratified tests showed significant group differences at 7–9 and 10–12 years but not at 13–14 years. Under low load, no significant group differences were detected in P3 amplitude or latency (all *p* > 0.05).

#### 3.2.4. Low-Load Condition Summary

Under low cognitive load, ANOVA and *t*-tests showed no significant group main effects or stable group interactions for P2, N2, or P3 (all *p* > 0.05). Only marginal between-group trends appeared in some age subgroups: P2 amplitude in 10–12 years (*p* = 0.07), N2 and P3 amplitude in 13–14 years (*p* = 0.071 and *p* = 0.070, respectively). None of these marginal effects reached statistical significance, confirming that group differences in electrophysiological markers were eliminated under low load.

## 4. Discussion

This study used a dual-task paradigm combined with high-temporal-resolution event-related potentials (ERPs) to investigate the age-related neurodevelopmental patterns of visuospatial attentional dysfunction in adolescents with ADHD across early perceptual and late cognitive control stages. Behavioral results confirmed that the ADHD group exhibited significantly lower accuracy and marginally prolonged reaction times relative to TD controls, particularly under high cognitive load. Convergent ERP evidence further revealed that ADHD-related deficits were primarily expressed in the late cognitive control stage indexed by P3 abnormalities, with more robust effects in the late stage, although early P2 effects were also observed under high load. These findings support the hypothesis that visuospatial attention impairment in ADHD is most prominently reflected in differences at the late-stage cognitive control and working-memory-related processes, although early perceptual differences were also observed under high load, with distinct age-related patterns across adolescent age groups. Importantly, the present study did not employ neuroimaging, functional connectivity analyses, or source localization techniques. Thus, our references to underlying neural networks are speculative inferences drawn from the previous literature and are not directly established by the current ERP data. Furthermore, because the present study employed a cross-sectional design to compare three independent age cohorts, all references to ‘developmental’ or ‘age-related’ effects in this discussion should be understood as describing cross-sectional differences between age groups, not as direct evidence of intra-individual change over time.

Consistent with prior behavioral studies [13], participants with ADHD showed poorer task performance under conditions requiring divided attention and working memory engagement. The low-load versus high-load contrast revealed robust load-dependent declines in accuracy, indicating that children with ADHD have particular difficulty allocating attentional resources efficiently when cognitive demands increase. This pattern aligns with the core executive dysfunction model of ADHD, which emphasizes impairments in sustained attention, inhibitory control, and working memory updating [14,15]. Together, these behavioral results demonstrate that ADHD is associated with a domain-general vulnerability in managing high-load visuospatial attention tasks, a pattern that has been previously linked to fronto-parietal network dysfunction in the broader literature [16]. The finding that accuracy, rather than mean RT, showed a robust group difference may suggest that children with ADHD prioritized maintaining performance at the expense of speed, or alternatively, that mean RTs are less sensitive than accuracy to the cognitive demands of this dual-task paradigm. This interpretation aligns with the view that ADHD-related performance deficits are more consistently captured by accuracy and variability measures than by mean speed alone.

Analysis of the P2 component revealed significant age-related effects on amplitude and latency, with shorter latencies in the ADHD group under high load. This pattern suggests altered early sensory gating and accelerated stimulus detection, possibly reflecting compensatory or dysregulated allocation of perceptual resources [17]. Age-group comparisons further indicated that P2 latency decreased progressively from childhood to early adolescence in both groups, reflecting typical maturational increases in information processing speed. However, the ADHD group showed atypical modulation of P2 by load, suggesting less flexible early attentional deployment during visuospatial processing. These results extend prior reports of altered early visual attention dynamics in ADHD [18] by demonstrating load-specific and age-dependent changes across adolescence.

In contrast, N2 amplitude and latency did not differ significantly between groups, suggesting relatively preserved response selection and conflict monitoring at this processing stage [5]. Although N2 is often linked to inhibitory control in ADHD [19], the absence of group differences in the current paradigm may reflect task-specific demands or compensatory neural recruitment during mid-level attentional processing. This null finding highlights the importance of distinguishing between perceptual selection, response inhibition, and late cognitive control when localizing ADHD-related attentional deficits.

The most robust group difference emerged in the P3 component: the ADHD group showed significantly greater P3 amplitude and longer latency under high cognitive load, with pronounced effects in the 7–9 and 10–12 year-old subgroups. Because P3 reflects context updating, working memory maintenance, and late-stage cognitive control [6,20], this pattern may reflect inefficient resource allocation and could be consistent with a compensatory neural response aimed at maintaining task performance. Prolonged P3 latency suggests delayed neural processing speed, consistent with the developmental lag hypothesis of ADHD [21,22]. Reduced P3 amplitude in previous studies was interpreted as diminished attentional resource allocation; however, the elevated amplitude seen here under high load could be interpreted as possible compensatory over-recruitment [23,24]. Although alternative interpretations—such as increased neural response variability, inefficient filtering of irrelevant inputs, or task-specific processing demands—cannot be excluded based on the present data. Future studies employing functional connectivity or source localization techniques are needed to distinguish among these possibilities. These P3 anomalies represent reliable neural markers of impaired late cognitive control in ADHD, particularly during visuospatial attention and working memory engagement.

Age-related differences in ERP measures further revealed age-dependent differences between groups. Latency measures of key ERP components decreased with age in TD children, indicating streamlined neural processing. In contrast, the ADHD group showed persistent latency prolongation and abnormal amplitude modulation, especially at younger ages, suggesting that the observed differences are not merely a delay but rather a persistent age-related gap between the groups at the time of measurement. These age-dependent differences are consistent with the prior neuroimaging literature suggesting the involvement of fronto-striatal–parietal circuits in ADHD [25,26]. By adolescence (12–14 years), between-group differences diminished, suggesting that between-group differences diminish with age at the group level, although the underlying mechanisms remain speculative and cannot be directly inferred from cross-sectional data.

### 4.1. Theoretical and Clinical Implications

Abnormal P3 under high cognitive load may be a promising candidate neurophysiological indicator. However, we emphasize that diagnostic utility (sensitivity, specificity) and predictive value were not evaluated in the present study; future ROC analyses and replications in independent samples are required before any clinical application can be considered. Interventions targeting working memory and executive control under high-load conditions may be particularly effective.

### 4.2. Limitations

Several limitations should be noted. First, the cross-sectional design cannot fully disentangle normative age-related changes from potential atypical patterns; longitudinal studies are needed. Second, the sample included children and adolescents aged 7–14 years; generalization to adult ADHD requires further testing. Additionally, the lack of a standardized clinical psychiatric interview for ADHD diagnosis, relying instead on parent-reported screening scales, limits the clinical generalizability of our findings. Future studies should incorporate structured diagnostic tools (e.g., K-SADS or MINI-KID) to confirm ADHD status. Third, the present study did not differentiate ADHD presentations (inattentive, hyperactive-impulsive, or combined). Although our sample comprised all three subtypes, the small number of participants in the hyperactive–impulsive subgroup precluded reliable statistical comparisons. Future research with larger sample sizes should investigate whether the observed P3 abnormalities are consistent across ADHD subtypes or reflect subtype-specific neurocognitive profiles. Fourth, the relatively small sample sizes within each age subgroup (approximately 20 participants per group) limit the statistical power for age-stratified comparisons and may have reduced our ability to detect subtle developmental interactions. Additionally, although sex distribution was balanced across groups, we did not formally test for sex × group interactions, which may be a relevant factor in ADHD neurophysiology. Fifth, our analysis of reaction times relied exclusively on mean values. Given that RT distributions in ADHD are often positively skewed, future research employing ex-Gaussian distribution fitting (estimating μ, σ, and τ parameters) may more precisely characterize the nature of RT slowing and variability in this population. Sixth, 95% confidence intervals were not reported for the principal effect sizes. While we provide exact *p*-values and partial *η*^2^ values for all main findings, the absence of CIs limits the direct evaluation of effect precision and clinical interpretability. Future studies should routinely report confidence intervals to facilitate the meta-analytic integration and clinical translation of ERP-based findings. Finally, reliance on parent-rated scales alone may introduce reporter bias; future work could integrate teacher ratings and clinical diagnostic interviews.

## 5. Conclusions

The present findings demonstrate that children and adolescents with ADHD show significant visuospatial attention deficits under high cognitive load, as indexed by both behavioral performance and ERP measures. While early perceptual processing (P2) shows load-dependent alterations, the most robust abnormalities were observed in the P3 component, supporting the view that late-stage cognitive control and working memory maintenance are particularly vulnerable in ADHD. P3 amplitude and latency abnormalities show clear age-related differences across childhood and early adolescence, suggesting developmental sensitivity of these neural measures. However, because our study is cross-sectional, these findings should be interpreted as age-related differences rather than direct evidence of age-related differences delay or deviance. Moreover, the diagnostic and clinical utility of P3 as a biomarker awaits future validation through longitudinal designs, ROC analyses, and intervention studies. Nevertheless, our results clarify the temporal dynamics of attentional dysfunction in ADHD and suggest that late-stage executive control processes may be promising targets for future interventional studies. However, the efficacy of any such intervention would need to be directly tested in controlled trials.

## Figures and Tables

**Figure 4:**
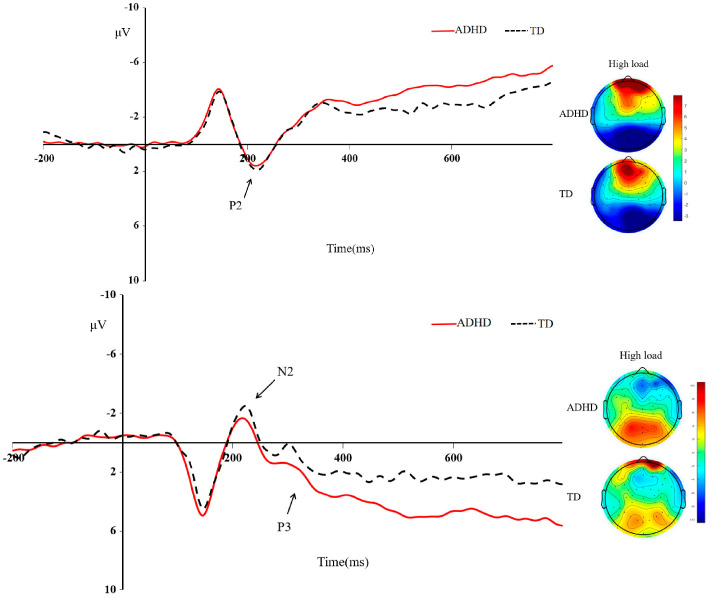
Waveform and topographic maps of the two groups of children under high-load conditions.

**Figure 5:**
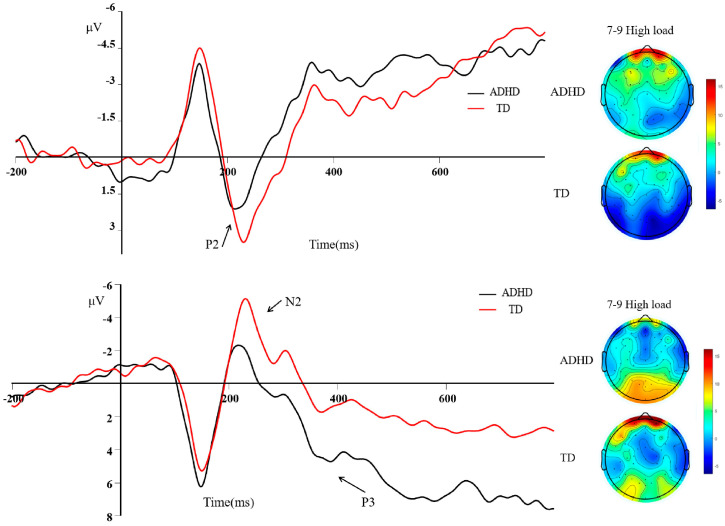
Waveform and topographic maps of two groups of subjects aged 7–9 years under high-load conditions.

**Figure 6:**
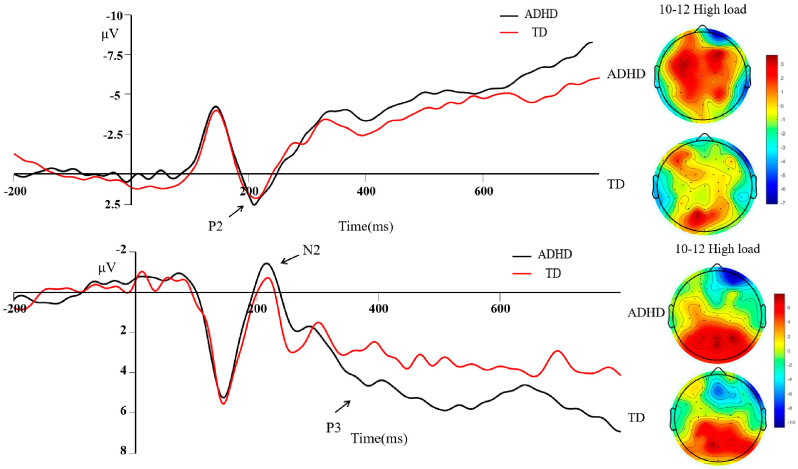
Waveform and topographic maps of two groups of subjects aged 10–12 years under high-load conditions.

**Figure 7:**
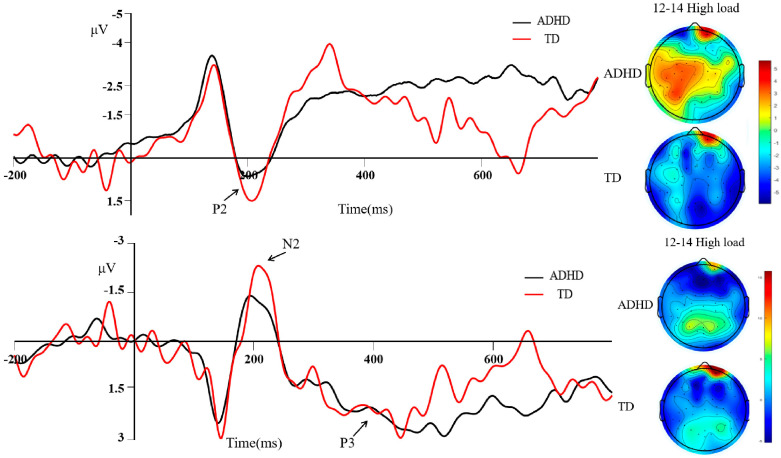
Waveform and topographic maps of two groups of subjects aged 12–14 years under high-load conditions.

**Figure 8:**
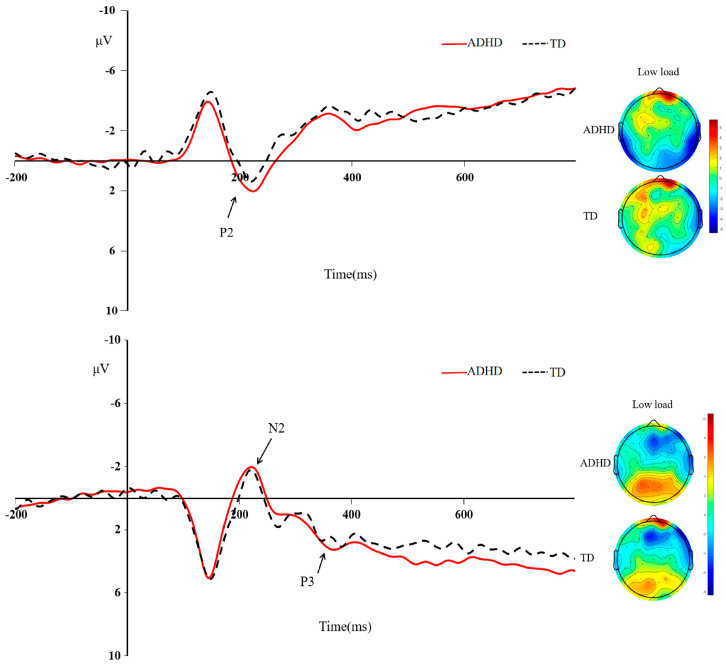
Waveform and topographic maps of the two groups of children under low-load conditions.

**Figure 9:**
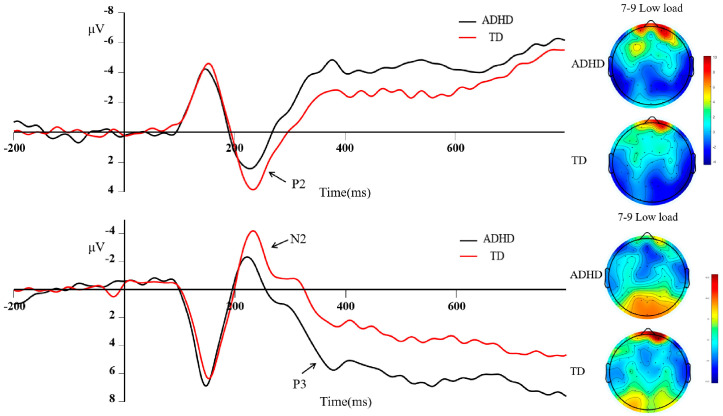
Waveform and topographic maps of two groups of subjects aged 7–9 years under low-load conditions.

**Figure 10:**
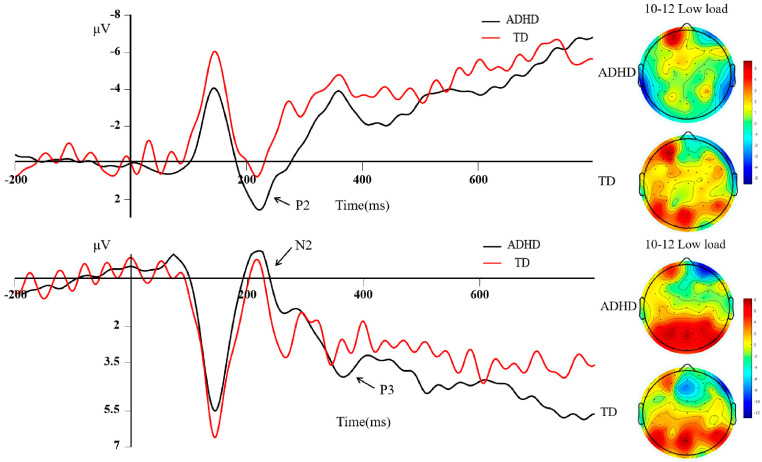
Waveform and topographic maps of two groups of subjects aged 10–12 years under low-load conditions.

**Figure 11:**
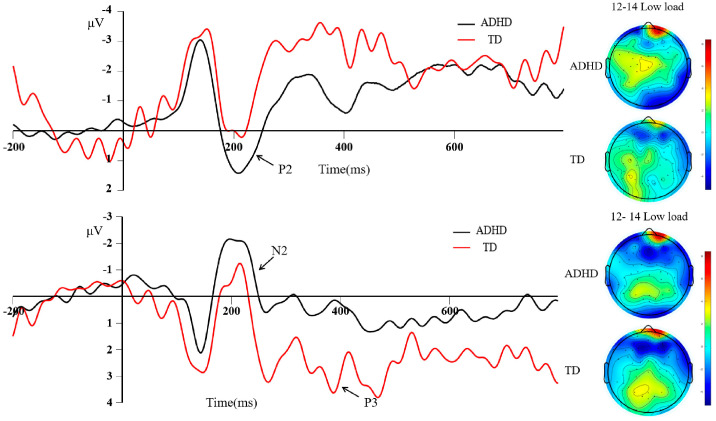
Waveform and topographic maps of two groups of subjects aged 12–14 years under low-load conditions.

**Table 1:** Demographic characteristics of participants.

	TD (N = 62)		ADHD (N = 62)	
Age Group	Boys:Girls	Age (M ± SD)	Boys:Girls	Age (M ± SD)
7–9	15:4	8.16 ± 0.74	15:4	7.89 ± 0.71
10–12	14:4	10.94 ± 0.73	13:6	10.57 ± 0.69
13–14	18:7	12.52 ± 0.51	17:7	12.71 ± 0.55

## Data Availability

The datasets generated during and/or analyzed during the current study are available from the corresponding author on reasonable request.
